# Women's Empowerment Dimensions and Child Stunting in Ethiopia: A Multilevel Analysis of Demographic and Health Surveys 2011–2016

**DOI:** 10.1111/mcn.70136

**Published:** 2025-11-29

**Authors:** Seyoum Teffera Mengesha, Eva Berde, Amare Zerihun Yohannes, Sándor Remsei

**Affiliations:** ^1^ Department of Economics College of Business and Economics Bahir Dar University Bahir Dar Ethiopia; ^2^ Institute of Economics Corvinus University of Budapest Budapest Hungary; ^3^ Széchenyi István University Győr Hungary; ^4^ Department of Disaster Risk Management & Sustainable Development Institute of Disaster Risk Management and Food Security Studies Bahir Dar University Bahir Dar Ethiopia

**Keywords:** child stunting, demographic health surveys, Ethiopia, regional heterogeneity, women's empowerment

## Abstract

Child stunting affects 38.3% of children under five in Ethiopia as of 2016. Women's empowerment, defined through both fundamental capabilities and household decision‐making authority, has emerged as a critical determinant of child nutritional outcomes. Drawing on Sen's capability approach and Kabeer's empowerment framework, we examined the associations between two distinct dimensions of women's empowerment and child stunting across Ethiopia's diverse regions.

We analyzed 18,466 mother–child pairs from the 2011 and 2016 Ethiopia Demographic and Health Surveys. Validated empowerment indices were constructed using factor analysis. We employed hierarchical multilevel models as our primary specification to examine the associations between women's empowerment and child stunting across Ethiopia's 11 administrative regions.

Between 2011 and 2016, stunting declined from 42.3% to 36.4%. Women's decision‐making authority increased (mean score: 0.70–0.78), while capabilities remained stable (0.17– 0.16). Higher capabilities were significantly associated with lower odds of stunting (*β* = −0.141, aOR = 0.87, 95% CI: 0.83, 0.91), whereas decision‐making showed no association (*β* = 0.013, aOR = 1.01, 95% CI: 0.98, 1.05). A significant interaction between capabilities and decision‐making was observed (*β* = 0.050, aOR = 1.05, 95% CI: 1.01, 1.09). Regional heterogeneity was substantial: Amhara maintained high stunting rates despite empowerment gains, while Somali saw improvements with low capabilities but increased decision‐making.

The study findings suggest that interventions should prioritize capability development through region‐specific strategies reflecting diverse pastoral, agrarian, and urban contexts; promote multi‐sectoral programs linking education and nutrition services; and develop monitoring frameworks to track both dimensions of empowerment at the regional level.

## Summary

- Women's capabilities (education, literacy, media access) show stronger associations with reduced child stunting than decision‐making authority (healthcare, purchases, mobility) across Ethiopian regions.
- Regional disparities persist with capability gaps remaining despite improved decision‐making authority, indicating uneven progress in women's empowerment dimensions.
- Effective nutrition interventions should prioritize capability strengthening alongside decision‐making authority, with region‐specific adaptations to address local contexts.

## Graphical abstract

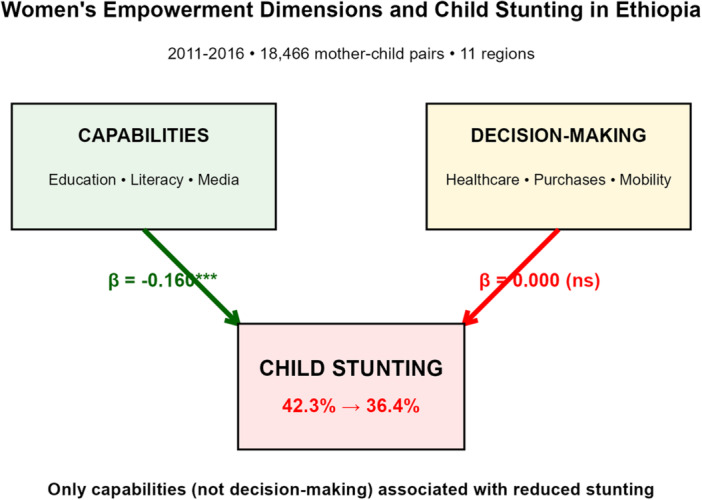

**Abbreviations**
AIC: akaike information criterion
aOR: adjusted odds ratio
BIC: bayesian information criterion
CDC: centers for disease control and prevention
CI: confidence interval
CSA: central statistical agency
DHS: demographic and health survey
EDHS: ethiopia demographic and health survey
EHNRI: ethiopia health and nutrition research institute
EPHI: ethiopian public health institute
FMoH: federal ministry of health
HAZ: height‐for‐age z‐score
ICF: inner city fund (formerly; now simply icf)
JMP: joint monitoring programme
MDE: minimum detectable effect
NCHS: national center for health statistics
NRERC: national research ethics review committee
OR: odds ratio
PNSP: productive safety net programme
PPS: probability proportional to size
PSU: primary sampling unit
RQ: research question
SDG: sustainable development goal
SE: standard error
UNICEF: united nations children's fund
WASH: water, sanitation, and hygiene
WHO: world health organization

## 1 Introduction

Child undernutrition, with stunting as one of its key indicators, remains a major global health concern, particularly in low‐ and middle‐income countries (Victora et al. 2021). Stunting is defined as a height‐for‐age Z score (HAZ) measurement more than two standard deviations below the World Health Organization (WHO) Child Growth Standards median (WHO 2006). Globally, it affects approximately 148.1 million children under 5 years of age, accounting for 22.3% of this population, despite recent efforts to reduce its prevalence (UNICEF, W., WB. 2023). The burden is disproportionately concentrated in Asia (52% of global cases) and Africa (43%), with sub‐Saharan Africa (SSA) facing the most severe challenges where stunting affects 41% of children under five, even amid regional economic growth (Quamme and Iversen 2022; UNICEF, W., WB. 2023).

Stunting is not only a health issue but also a profound economic concern. It compromises cognitive development, educational achievement, and future productivity, thus perpetuating intergenerational cycles of poverty. Stunted children tend to become less productive adults. Conversely, investments in stunting prevention yield high returns, with benefit‐cost ratios ranging from 3.6 in the Democratic Republic of Congo to 48 in Indonesia, and a median of 18 in Bangladesh, making nutrition interventions among the most cost‐effective development strategies available (Hoddinott et al. 2013).

This economic imperative is particularly relevant in Ethiopia, a country that presents a stark paradox. Despite averaging 9.1% annual economic growth between 2000/01 and 2016/17 (World Bank 2020), the country still struggles with high rates of chronic malnutrition: 38.3% of children under five are stunted (CSA, & ICF. 2016). This persistent nutritional challenge amid economic growth suggests that determinants beyond macroeconomic indicators, including household dynamics and individual empowerment factors play crucial roles in determining children's nutritional outcome.

In this context, women's empowerment emerges as a pivotal factor, given that mothers are the primary caregivers responsible for children's diets and healthcare access in Ethiopia (Christian et al. 2023). However, despite its recognized importance, the multidimensional nature of women's empowerment and its nuanced relationship with child nutrition remains insufficiently explored, especially across Ethiopia's diverse regions.

Our study addresses this gap by examining how different dimensions of women's empowerment are associated with child stunting in Ethiopia using data from the 2011 and 2016 Demographic and Health Surveys. We conceptualize women's empowerment through two distinct but interconnected dimensions: capabilities (education, literacy, media access) and decision‐making authority (regarding healthcare, purchases, mobility). This dual conceptualization draws on Sen's capability approach, which distinguishes between formal freedoms and substantive capabilities—the actual abilities individuals possess to achieve valued outcomes (Sen 1999), and Kabeer's framework, emphasizing that meaningful empowerment requires the integration of decision‐making authority with resources and agency (Kabeer 1999).

The theoretical associations between women's empowerment dimensions and child stunting operate through distinct mechanisms. Capabilities may affect child nutrition through knowledge and resource access pathways: educated women better understand nutritional needs, interpret health information, and navigate healthcare systems (Alderman and Headey 2017). Literacy enables comprehension of feeding guidelines and medication instructions, while media exposure provides awareness of best practices in childcare and nutrition (Smith et al. 2003). Through these knowledge pathways, capabilities may facilitate improved feeding practices, timely healthcare seeking, and better hygiene behaviors.

Decision‐making authority may function through resource control and time allocation pathways. Women with household decision‐making power can direct resources toward child health and nutrition, influence food purchasing decisions, and allocate time for childcare activities (Carlson et al. 2015). However, decision‐making without corresponding capabilities may be ineffective, as research demonstrates that empowerment operates through enabling women to leverage available resources and knowledge (Heckert et al. 2019).

The interaction between these dimensions suggests complementary effects: capabilities provide the knowledge base for informed decisions, while decision‐making authority enables implementation of this knowledge. This synergy may be particularly important in contexts where traditional gender norms limit women's agency despite formal education (Jayachandran 2015). The relevance of this dual framework becomes particularly evident when examining Ethiopian women's empowerment, where our analysis of CSA, & ICF. (2016) data show that while 71% of women report participating in household decision‐making, only 20% have completed primary education. This gap between decision‐making involvement and fundamental capabilities may help explain persistent child nutrition challenges in contexts where patriarchal household dynamics and traditional gender norms limit women's access to education and information (Christian et al. 2023). Moreover, Ethiopia's diverse regional landscape, ranging from urban centers with relatively higher female literacy to rural and pastoral areas with deeply entrenched traditional gender norms (UN Women, & European Union. 2014), provides a unique opportunity to examine how varying combinations of capabilities and decision‐making authority are associated with child nutrition across different sociocultural contexts.

Previous research has established important connections between women's empowerment and child nutrition outcomes. In India, Shroff et al. (2009) found maternal autonomy inversely associated with stunting, while Richardson (2018) emphasized the importance of context‐specific approaches to measuring empowerment across diverse settings. Studies in sub‐Saharan Africa have demonstrated significant associations between various empowerment measures and childhood nutritional status. Yaya et al. (2020) examined this relationship across 30 countries in the region, finding significant associations between decision‐making, attitudes toward violence, and experience of violence with childhood nutritional status after controlling for other covariates. In Ethiopia specifically, Mekonnen et al. (2021) identified three key domains—attitude towards violence, social independence, and decision‐making—as significantly associated with reduced stunting and underweight. More recently, Wassie et al. (2024) found that mothers with moderate or high empowerment had lower odds of having stunted children compared to those with low empowerment.

While these studies provide valuable insights, existing research has primarily used single dimensions of empowerment, cross‐sectional data, or analyses limited to either national aggregates or specific sub‐regions. This limits our understanding of how different empowerment dimensions interact to affect child nutrition across diverse contexts. Our study addresses these limitations by applying a multidimensional framework specifically tailored to Ethiopia's sociocultural context, examining both independent and interactive effects of women's capabilities and decision‐making authority on child stunting. While we acknowledge the cross‐sectional and observational nature of our data limits causal inference, our analysis provides important insights into these associations across Ethiopia's diverse regions.

Our study period of 2011–2016 provides additional analytical value, as it captures outcomes following Ethiopia's implementation of the National Nutrition Program and Growth and Transformation Plans, which explicitly targeted both women's empowerment and child nutrition (FDRE Ministry of Finance and Economic Development 2013). The National Nutrition Program (2013–2015) included specific components for maternal education and women's empowerment through health extension workers, while the Growth and Transformation Plan I (2010–2015) emphasized gender equality in its development objectives (FDRE Ministry of Finance and Economic Development 2010). By analyzing two consecutive nationally representative surveys from this critical policy implementation period, we address the following interconnected research questions:

**Primary Research Question**: What are the independent and interactive associations between women's capabilities and decision‐making authority with child stunting in Ethiopia, and how have these relationships changed between 2011 and 2016?

**Secondary Research Question**: How do these associations vary across Ethiopia's regions and between rural and urban settings?

By addressing these questions, our study contributes to both the theoretical understanding of empowerment‐nutrition pathways and provides practical insights for nutrition and gender‐focused interventions in Ethiopia and similar contexts.

## 2 Methods

### 2.1 Data Source and Study Population

The Ethiopia Demographic and Health Survey (EDHS) is a nationally representative household survey that provides data on key population, health, and nutrition indicators. It is conducted by the Central Statistical Agency (CSA) in collaboration with the Federal Ministry of Health (FMoH) and the Ethiopian Public Health Institute (EPHI), with technical assistance from ICF International and financial as well as technical support from development partners (CSA, & ICF. 2016). The DHS survey in general employs standardized data collection methods across countries, making it an ideal tool for cross‐national comparisons and time‐series analyses of developmental trends. For this study, we analyzed data from the 2011 and 2016 EDHS, selecting these waves for their comparable survey design, methodological consistency, and data quality in addition to the policy implementation justification we mentioned in the introduction.^1^

Both the 2011 and 2016 waves of the EDHS utilized a consistent two‐stage stratified cluster sampling design to ensure national representativeness. In the first stage, enumeration areas (EAs) were selected using probability proportional to size (PPS) based on the 2007 Population and Housing Census. In the second stage, households were systematically selected from a complete listing within each selected EA. The country was stratified first by region and then by urban and rural areas within each region, forming distinct sampling strata. The 2011 EDHS included 23 sampling strata and 596 enumeration areas (clusters), while the 2016 EDHS expanded to 25 strata and 643 clusters, reflecting updates to the sampling frame and regional classifications. Both surveys maintained consistency in key design variables, including cluster identifiers, stratification variables, and sampling weights to facilitate accurate analysis and comparability over time (CSA, & ICF, & ICF 2012, 2016).

We applied the following inclusion criteria: mother‐child pairs where the child was under 5 years old and had valid anthropometric measurements. Values of height‐for‐age z‐scores (HAZ) ≥ 9996, representing measurements outside biologically plausible ranges (typically beyond ±6 standard deviations), were excluded following standard DHS protocols (MEASURE DHS 2012). Cases with missing but non‐flagged HAZ values were retained for multiple imputation. To assess potential selection bias arising from missing data, we compared characteristics between included and excluded observations (Appendix Table A2). For 2011, valid anthropometric cases versus those without showed nearly identical capability scores (0.162 vs. 0.161) and comparable decision scores (0.677 vs. 0.649). The 2016 wave demonstrated even more consistent patterns across capability scores (0.177 vs. 0.164), decision scores (0.779 vs. 0.778), and urban residence proportions (18.4% vs. 19.5%). For descriptive statistics, we used complete‐case data with non‐missing values on all relevant variables, while regression models employed multiple imputations to handle partial missingness while maintaining the sample size of eligible mother–child pairs.

### 2.2 Variable Definitions

Our primary outcome variable was stunting. We focused on stunting for three key reasons: First, stunting represents chronic undernutrition and better reflects long‐term socioeconomic and care practices, making it most relevant for assessing women's empowerment effects. Second, our preliminary analyses showed that because wasting remained relatively stable between waves (9.9%–10.1%), underweight (a composite index of stunting and wasting) trends (28.8%–23.3%) closely mirrored stunting patterns (44.3%–38.3%), with similar associations to empowerment measures. Third, stunting's high prevalence and substantial regional variation provided greater statistical power for detecting empowerment‐nutrition relationships across Ethiopia's diverse regions.

To examine how women's empowerment influences these stunting patterns, we developed measurement approaches that capture empowerment's multidimensional nature. Based on Sen's capability approach (Sen 1999) and Kabeer's empowerment framework (Kabeer 1999), we constructed two distinct indices operationalizing these theoretical dimensions. For a detailed explanation of the construction, validation, and psychometric properties of these indices, please refer to Appendix Tables A3, A4, A5. The capability index measured fundamental abilities through three weighted components: educational attainment (50%) scored on a four‐point scale (none = 0, primary = 0.33, secondary = 0.67, higher = 1.0), literacy status (30%) scored as 0 (cannot read), 0.5 (reads with difficulty), or 1.0 (reads easily), and media exposure (20%). Media exposure was calculated as the mean of three binary indicators representing access to newspapers, radio, and television, where each medium was coded as one if the woman reported using it at least once a week and zero otherwise. This created a composite media access score ranging from 0 (no access) to 1 (access to all three media types). Note that Tables 3A and 3B in the results section present the original DHS coding of decision‐making variables (which ranges from 1 = woman alone to 4 + = others) to show regional patterns in raw responses, while our index construction uses the recoded binary indicators described here.

The decision‐making index assessed household‐level agency through three weighted domains: healthcare decisions (40%), large household purchases (40%), and mobility choices (20%). Each domain was coded as 1 for woman alone/joint decision or 0 for husband alone/others. The 40:60 weighting ratio between capability and decision‐making dimensions was derived from exploratory factor analysis, which showed approximately 40% of empowerment variance was captured by capability items and 60% by decision‐making items (see Appendix Table A3). Measurement reliability was evaluated using multiple methods (Appendix Table A4). To ensure robustness, the weighting structure was further validated through sensitivity analyses using six alternative schemes, all of which yielded consistent patterns (Appendix Table A5). Additionally, we conducted a minimum detectable effects (MDE) analysis to evaluate regional statistical power (Appendix Table A6).

For regression analyses, we standardized both capability and decision‐making indices to have mean of 0 and standard deviation of 1 to facilitate comparison of effect sizes. Before standardization, we transformed raw DHS variables by recoding nominal categorical variables into binary or ordinal scales as appropriate (e.g., decision‐making responses were recoded from categorical responses to binary indicators of woman's participation).

In addition to our empowerment measures, control variables were selected based on established determinants of child stunting and guided by conceptual frameworks of undernutrition. We included maternal factors (age in 5‐year groups from 15 to 49 years), child characteristics (age, sex, birth order), household socioeconomic status (wealth quintiles derived following standard DHS methodology (Rutstein and Johnson 2004)), environmental factors (Water, Sanitation, and Hygiene [WASH] index adapted from WHO/UNICEF Joint Monitoring Programme [JMP] classifications (UNICEF, & WHO. 2017)), residential context (urban/rural), and regional indicators to account for geographical heterogeneity across Ethiopia's 11 administrative regions (Bitew et al. 2023). Birth order was included as a control variable based on systematic review evidence identifying it among the most consistent factors associated with child malnutrition (Katoch 2022). Ethiopia's regions reflect diverse livelihood systems such as agrarian highlands (Amhara, Tigray, large parts of Oromia), pastoral lowlands (Afar and Somali), and urban centers (Addis Ababa, Harari and Dire Dawa).

### 2.3 Statistical Analysis

To address the complex sampling structure of EDHS, we implemented a survey‐weighted analytical strategy following the Rutstein and Johnson (2004) methodological guidelines. We developed four models to examine different aspects of the empowerment‐stunting relationship, with our primary analysis focusing on the hierarchical capability‐decision interaction model that best captures the complex relationships between empowerment dimensions and child stunting.

Our main analytical model therefore is:

$$\begin{array}{rcl} logit\left(P\left({\mathrm{stunting}}_{ir}\right)\right) & = & \alpha +\beta_{1}\mathrm{Capability}+\beta_{2}\mathrm{Decision}\\ & & +\;\beta_{3}\mathrm{Wave}+\beta_{4}(\mathrm{Capability}\times \mathrm{Decision})\\ & & +\;\gamma \;\mathrm{Controls}+u_{r} \end{array}$$

where $\beta_{1}$ represents the effect of the capability index, $\beta_{2}$ represents the effect of the decision‐making index, $\beta_{3}$ represents the effect of the survey wave, $\beta_{4}$ represents the interaction effects between capability and decision‐making, γ represents coefficients for control variables, and $u_{r}$ represents the random intercept for region r, allowing baseline stunting probability to vary by region while maintaining fixed effects for our primary predictors.

To ensure robustness, we also estimated three complementary models: survey‐weighted main effects and wave interaction models (Models A1‐A2), and a hierarchical main effects model (Model A3) (See Appendix Models).

Model implementation varied according to design requirements. For survey‐weighted models, we incorporated DHS complex survey design using the “survey” package in R, which accounts for sampling weights (v005/1000000), clustering (v001), and stratification (v023) in both point estimates and standard errors. We used quasibinomial logistic regression to account for potential overdispersion in binary outcomes (Goldstein 2010). For hierarchical models, we implemented a multilevel approach using the “lme4” package, with regions as the higher‐level grouping variable. We examined the capability–decision interaction using our primary hierarchical model (Table 2). Although hierarchical models do not directly incorporate sampling weights, they account for the geographical clustering inherent in the survey design by including random effects. To complement this model, we also tested alternative interaction specifications, including interactions with survey wave (time effects), in survey‐weighted models (Appendix Table A10). These additional models provide supplementary evidence alongside the capability–decision interaction analyses.

Missing data handling followed established best practices. To handle missing data in predictor variables (while maintaining our sample of eligible mother‐child pairs with valid anthropometric measurements), we employed multiple imputation using predictive mean matching with 20 imputed datasets and 30 iterations, following recommended practices for complex survey designs (van Buuren and Groothuis‐Oudshoorn 2011). We included all analysis variables plus auxiliary predictors related to missingness patterns in the imputation model. Parameter estimates and standard errors were pooled across imputed datasets using Rubin's rules (Rubin 1976).

Missing data patterns varied by domain (Appendix Table A7). Anthropometric data showed substantial missingness (~17%), split between flagged values and true missing. Decision‐making variables showed identical missingness patterns within each wave. All capability and demographic variables had complete data.

Model evaluation combined multiple criteria. Model selection utilized both Akaike Information Criterion (AIC) and Bayesian Information Criterion (BIC) to balance fit and parsimony, while methodological rigor was enhanced through power analyses and calculation of MDEs by region (Appendix Table A9). The regional MDEs, which represent the smallest effect sizes that can be reliably detected given the sample size and statistical power, ranged from 0.693 to 1.08 for decision‐making outcomes and from 0.695 to 0.965 for anthropometric measurements. These ranges indicate that the study had adequate power to detect moderate effects in most regions. All regression model results are presented as adjusted odds ratios (AOR) with 95% confidence intervals (95% CI) to facilitate interpretation of effect sizes.

All analyses were implemented in R (version 4.5.0) (R Core Team 2025), using specialized packages including “survey” for complex survey design analysis (Lumley 2004), “lme4” for hierarchical modeling (Bates et al. 2025), “mice” for multiple imputation (van Buuren and Groothuis‐Oudshoorn 2011), and “psych” for measurement validation (Revelle 2025).

### 2.4 Ethics Approval and Consent to Participate

The 2011 EDHS received ethical approval from the Ethiopia Health and Nutrition Research Institute (EHNRI) Review Board, the National Research Ethics Review Committee (NRERC) at the Ministry of Science and Technology, the Institutional Review Board of ICF International, and the U.S. Centers for Disease Control and Prevention (CDC). The 2016 EDHS received approval from the Federal Democratic Republic of Ethiopia Ministry of Science and Technology and the Institutional Review Board of ICF International. This secondary analysis, which utilized publicly available and deidentified data, was exempt from further ethical review. Access to the data set was granted by the appropriate data providers following registration of the research project. The EDHS data collection procedures and ethical considerations are detailed in the official survey reports (CSA, & ICF, & ICF. 2012, 2016).

## 3 Results

### 3.1 Sample Characteristics and Data Quality

**Figure 1:**
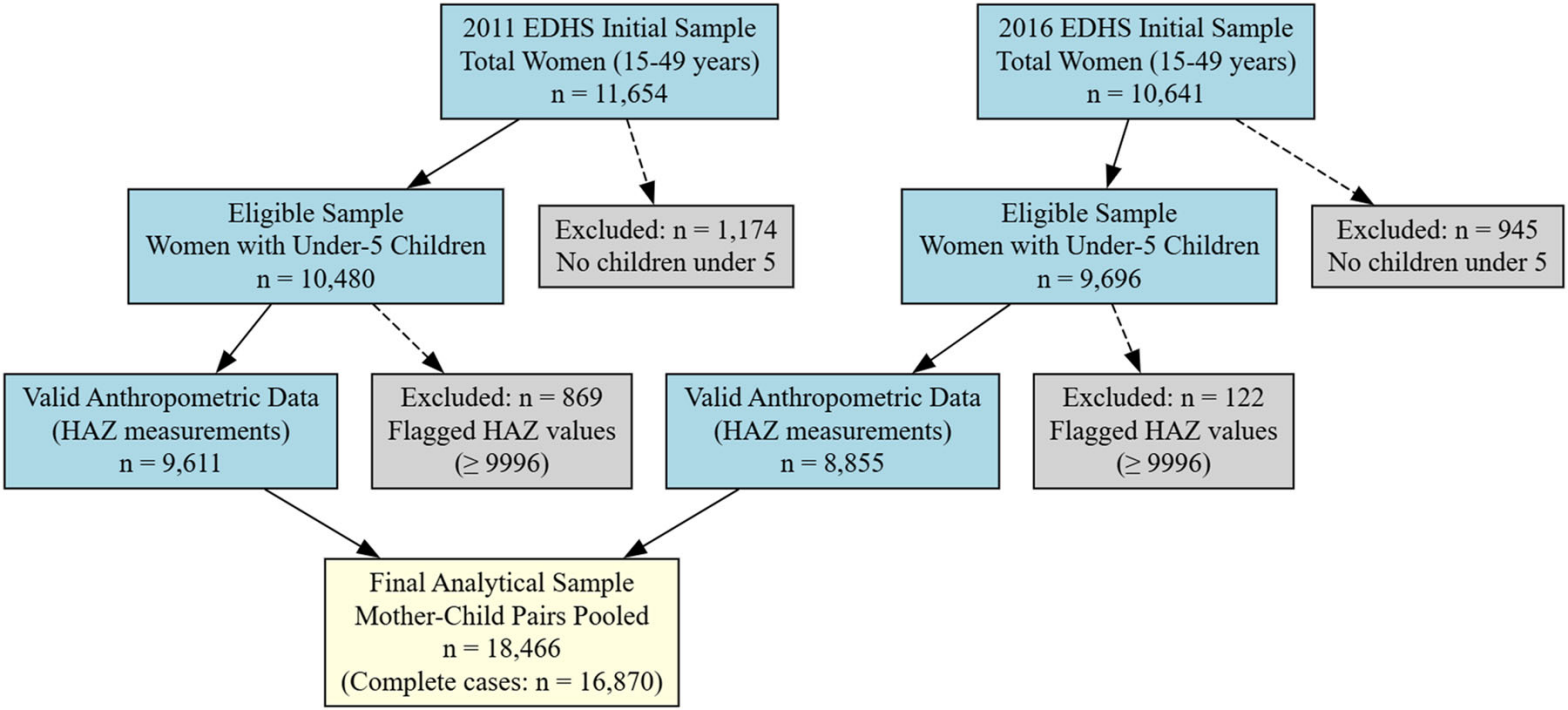
Flow diagram showing sample selection process.

From the initial samples of 11,654 women (2011) and 10,641 women (2016), our selection criteria yielded a final analytical sample of 18,466 mother–child pairs. We first restricted analysis to women with children under 5 years (10,480 in 2011, 89.9%; 9696 in 2016, 91.1%). After excluding observations with flagged anthropometric values, we included 9611 pairs from 2011 (82.5% of the initial sample) and 8855 pairs from 2016 (83.2%). For descriptive analyses requiring complete data on all variables, the sample comprised 8645 pairs from 2011 (74.2%) and 8225 pairs from 2016 (77.3%), totaling 16,870 complete mother‐child pairs [see Figure 1 for selection process].

Our analytical sample captured important demographic transitions between survey waves. Table 1 summarizes these key temporal changes in sample characteristics.

**Table 1:** Sample characteristics, 2011–2016.

Characteristic	2011	2016
Residence type (%)		
Urban	17.0	18.6
Rural	83.0	81.4
Maternal education (%)		
No education	66.3	61.8
Primary	27.1	30.2
Secondary+	6.6	8.0
Maternal age (%)		
15–24 years	24.0	22.2
25–34 years	51.7	53.0
35–49 years	24.2	24.8
Mean parity	4.2	4.3
Child age distribution (%)		
0–11 months	20.0	20.5
12–59 months	70.6	71.6
No measurements	9.4	7.9
Birth order (%)		
First	19.8	20.4
Second	17.4	16.9
Third	14.5	14.4
Fourth	12.5	12.4
Fifth+	35.8	35.8
Child sex distribution (%)		
Male	50.9	50.9
Female	49.1	49.1
Mean WASH index (1–3 scale)	1.59	1.72

Notable demographic shifts emerged between 2011 and 2016. The urban population increased modestly from 17.0% to 18.6%, while the proportion of women without formal education decreased from 66.3% to 61.8%. The proportion of women aged 25–34 expanded from 51.7% to 53.0%, and the mean parity increased slightly from 4.2 to 4.3. Environmental conditions improved, as indicated by the rise in the WASH index from 1.59 to 1.72.

Geographic distribution analysis revealed both demographic shifts and systematic patterns in data completeness (Appendix Table A8). The Amhara region sample share declined from 11.1% to 9.2%, while the Somali region increased from 8.8% to 14.1%. Pastoral regions showed consistently higher rates of missing anthropometric data, with missingness in Afar increasing from 13.7% to 23.1% and in Somali from 12.5% to 20.9%, while Tigray maintained relatively stable rates (8.2%–8.5%). These demographic patterns provide important context for understanding how empowerment dimensions may differentially affect child nutrition across population subgroups.

With this comprehensive understanding of our sample's characteristics and representativeness, we turn to examining the relationship between women's empowerment and child stunting.

#### 3.1.1 RQ1: Associations Between Women's Capabilities, Decision‐Making and Child Stunting

The primary analysis employed a hierarchical interaction model that best captured the complex associations between empowerment dimensions and child stunting. Table 2 presents results from this optimal specification, which accounts for regional clustering through random effects.

**Table 2:** Hierarchical interaction model of child stunting.

Variable	Coefficient (SE)	aOR	95% CI	*p*‐value
Intercept	0.170 (0.154)	1.19	(0.88, 1.60)	0.270
Capability index	−0.141 (0.021)	0.87	(0.83, 0.91)	< 0.001***
Decision index	0.013 (0.016)	1.01	(0.98, 1.05)	0.437
Wave (2016 vs. 2011)	−0.164 (0.033)	0.85	(0.80, 0.91)	< 0.001***
Maternal age (ref: 15–19)				
20–24	−0.083 (0.090)	0.92	(0.77, 1.10)	0.360
25–29	−0.090 (0.094)	0.91	(0.76, 1.10)	0.337
30–34	−0.141 (0.100)	0.87	(0.71, 1.06)	0.159
35–39	−0.119 (0.104)	0.89	(0.72, 1.09)	0.255
40–44	−0.172 (0.115)	0.84	(0.67, 1.05)	0.136
45–49	−0.376 (0.144)	0.69	(0.52, 0.91)	0.009**
Child age (ref: 24–59 m)				
0–5 m	−1.639 (0.061)	0.19	(0.17, 0.22)	< 0.001***
6–11 m	−1.409 (0.061)	0.24	(0.22, 0.27)	< 0.001***
12–23 m	−0.247 (0.039)	0.78	(0.72, 0.85)	< 0.001***
Birth order (ref: Fifth + )				
First	0.098 (0.070)	1.10	(0.96, 1.26)	0.159
Second	−0.113 (0.058)	0.89	(0.80, 1.00)	0.049*
Third	−0.055 (0.053)	0.95	(0.85, 1.05)	0.299
Fourth	−0.013 (0.051)	0.99	(0.89, 1.09)	0.801
Wealth (ref: poorest)				
Poorer	−0.059 (0.050)	0.94	(0.85, 1.04)	0.239
Middle	−0.100 (0.050)	0.90	(0.82, 1.00)	0.046*
Richer	−0.263 (0.053)	0.77	(0.69, 0.85)	< 0.001***
Richest	−0.485 (0.073)	0.62	(0.53, 0.71)	< 0.001***
Residence (ref: Urban)				
Rural	0.103 (0.067)	1.11	(0.97, 1.27)	0.121
Capability × decision	0.050 (0.020)	1.05	(1.01, 1.09)	0.011*
Random effects				
Region variance	0.084	—	—	—

*Note:* Dependent variable is stunting status. Significance levels.

Model fit: Hierarchical models showed better fit (main effects AIC: 22360; interaction AIC: 22355) compared to survey‐weighted models (main effects AIC: 23042; interaction AIC: 23048).

* *p* < 0.05

** *p* < 0.01

*** *p* < 0.001.

The hierarchical interaction model reveals three key findings. First, the capability index demonstrated a significant negative association with stunting (*β* = −0.141, aOR = 0.87, 95% CI: 0.83, 0.91), with each unit increase in capabilities associated with 13% lower odds of stunting. Second, the decision‐making index showed no significant independent association with stunting (*β* = 0.013, aOR = 1.01, 95% CI: 0.98, 1.05). Third, the capability‐decision interaction term was statistically significant (*β* = 0.050, aOR = 1.05, 95% CI: 1.01, 1.09), indicating that the protective association of capabilities diminishes as decision‐making authority increases, suggesting these empowerment dimensions may operate as substitutes rather than complements.

The wave effect demonstrates consistent improvement in stunting outcomes between 2011 and 2016 (*β* = −0.164, aOR = 0.85, *p* < 0.001), with 15% lower odds of stunting in the later wave. Among control variables, child age emerged as the strongest correlate, with younger children showing substantially lower stunting prevalence: 0–5 months (aOR = 0.19), 6–11 months (aOR = 0.24), and 12–23 months (aOR = 0.78) compared to children aged 24–59 months. Household wealth demonstrated clear socioeconomic gradients, with richer (aOR = 0.77) and richest quintiles (aOR = 0.62) showing significant negative associations with stunting. Maternal age showed a protective association only for mothers aged 45–49 (aOR = 0.69, *p* = 0.009), while other age groups demonstrated nonsignificant trends toward lower stunting odds.

These associations remained robust across alternative model specifications. Appendix Table A9 presents survey‐weighted main effects results, confirming the negative capability association (*β* = −0.154, *p* < 0.001) and null decision‐making association (*β* = 0.018, *p* = 0.483). Regional analysis reveals substantial disparities compared to Addis Ababa: Amhara (aOR = 2.10), Tigray (aOR = 1.83), Benishangul‐Gumuz (aOR = 1.71), Afar (aOR = 1.59), SNNPR (aOR = 1.52), Dire Dawa (aOR = 1.46), and Oromia (aOR = 1.39). Appendix Table A10 demonstrates temporal stability in the capability‐stunting association (capability × wave interaction: *β* = −0.094, *p* = 0.082). Appendix Table A11 reinforces the capability's negative association (*β* = −0.153, *p* < 0.001) and confirms substantial regional heterogeneity (region variance = 0.084).

The regional random effects component (variance = 0.084) justified the hierarchical approach and indicated meaningful variation in baseline stunting prevalence across Ethiopia's regions, even after controlling for individual and household characteristics.

Finally, we examine how these relationships evolved during our study period (2011–2016). Ethiopia experienced significant improvements in child nutritional status between 2011 and 2016. National stunting prevalence decreased from 42.3% to 36.4%, while mean HAZ improved from −1.61 to −1.36. Women's empowerment dimensions showed contrasting patterns (Appendix Table A12). The capability index declined slightly from 0.17 to 0.16, driven primarily by decreased media exposure (0.30–0.17), despite gains in education (0.12–0.14) and literacy (0.17–0.18). Conversely, decision‐making authority strengthened substantially from 0.70 to 0.78, with improvements in healthcare (0.72–0.79), purchases (0.64–0.76), and mobility decisions (0.76 –0.82). This temporal strengthening suggests that capabilities became increasingly important for child nutrition as Ethiopia developed. The national‐level patterns we have identified may mask important regional variations, leading us to examine geographic heterogeneity in empowerment‐stunting relationships.

#### 3.1.2 RQ2: Regional Variations in Empowerment‐Stunting Relationships

Regional analysis revealed substantial heterogeneity in both nutritional outcomes and empowerment dimensions across Ethiopia's 11 regions from 2011 to 2016. Tables 3A and 3B present comprehensive regional comparisons of stunting prevalence, mean HAZ scores, and empowerment indicators across both survey waves.

**Table 3A:** Regional child nutrition outcomes and overall empowerment (2011–2016).

	Stunting (%)		HAZ		Capability index		Decision index	
Region	2011	2016	2011	2016	2011	2016	2011	2016
Amhara	51.80	46.80	−2.02	−1.81	0.12	0.14	0.74	0.86
Tigray	51.70	39.70	−2.01	−1.57	0.19	0.24	0.79	0.83
Benishangul‐Gumuz	49.30	44.00	−1.96	−1.78	0.11	0.13	0.62	0.74
Afar	51.30	43.80	−1.87	−1.68	0.07	0.07	0.63	0.70
SNNPR	44.10	38.50	−1.71	−1.44	0.16	0.16	0.63	0.74
National	42.30	36.40	−1.61	−1.36	0.16	0.18	0.68	0.78
Oromia	41.00	36.00	−1.52	−1.34	0.18	0.14	0.71	0.78
Dire Dawa	36.60	40.00	−1.45	−1.34	0.16	0.22	0.73	0.82
Somali	32.80	27.20	−1.12	−0.84	0.08	0.06	0.47	0.72
Harari	29.00	31.70	−1.09	−1.15	0.25	0.27	0.73	0.91
Gambela	27.40	25.90	−0.94	−0.97	0.15	0.25	0.63	0.70
Addis Ababa	21.70	14.60	−0.86	−0.59	0.57	0.69	0.89	0.91

**Table 3B:** Components of women's empowerment dimensions by region (2011–2016).

Region	Capability components						Decision components					
	Education		Literacy		Media		Health		Purchase		Mobility	
	2011	2016	2011	2016	2011	2016	2011	2016	2011	2016	2011	2016
Amhara	0.07	0.11	0.14	0.20	0.22	0.13	2.34	2.19	2.65	2.25	2.28	2.09
Tigray	0.13	0.20	0.22	0.31	0.30	0.22	2.11	2.08	2.66	2.29	2.19	2.20
Benishangul‐Gumuz	0.09	0.14	0.10	0.14	0.17	0.10	2.60	2.32	2.79	2.57	2.57	2.21
Afar	0.04	0.06	0.06	0.07	0.19	0.10	2.51	2.47	2.79	2.52	2.40	2.37
SNNPR	0.13	0.17	0.14	0.16	0.27	0.15	2.55	2.32	2.91	2.48	2.36	2.31
National	0.12	0.17	0.16	0.20	0.27	0.17	2.44	2.25	2.71	2.39	2.36	2.16
Oromia	0.12	0.12	0.17	0.15	0.34	0.16	2.51	2.35	2.59	2.42	2.37	2.23
Dire Dawa	0.12	0.20	0.14	0.23	0.26	0.23	2.30	2.20	2.48	2.24	2.43	1.89
Somali	0.06	0.07	0.08	0.07	0.17	0.07	2.87	2.23	3.08	2.47	2.73	1.95
Harari	0.19	0.25	0.23	0.28	0.42	0.29	2.26	2.02	2.60	2.11	1.92	2.03
Gambela	0.19	0.32	0.13	0.25	0.14	0.13	2.47	2.27	2.70	2.47	2.42	2.25
Addis Ababa	0.44	0.59	0.69	0.83	0.72	0.71	1.91	1.85	2.14	2.08	1.80	1.81

*Note:* Regions ordered by 2011 HAZ scores (worst to best). These Tables present original DHS coding of variables to show regional patterns, while standardized values used in statistical models are described in the Variable Definitions section. Some regions show contradictory trends between HAZ and stunting due to distributional changes around the stunting threshold (−2 SD).

While these aggregate figures offer valuable insight, they obscure important distributional nuances, particularly where stunting prevalence and mean HAZ moved in opposite directions. In several regions, divergent trends between HAZ and stunting prevalence were observed, largely attributable to threshold effects around the stunting cutoff point. In Gambela, for instance, mean HAZ declined slightly from –0.94 to –0.97, yet stunting prevalence improved from 27.4% to 25.9%. Similarly, Harari experienced a modest decline in mean HAZ from –1.09 to –1.15, accompanied by an increase in stunting prevalence from 29.0% to 31.7%. In contrast, Dire Dawa recorded an increase in mean HAZ from –1.45 to –1.34, yet saw an unexpected rise in stunting prevalence from 36.6% to 40.0%. Verification analyses confirmed that between 3% and 6% of children in these regions had HAZ scores narrowly clustered around the –2.0 threshold, specifically between –2.1 and –1.9, making stunting classification highly sensitive to small shifts in the underlying distribution.

Addis Ababa consistently reported the most favorable outcomes over the study period. Mean HAZ improved from –0.86 to –0.59, while stunting prevalence declined from 21.7% to 14.6%. These improvements coincided with the highest observed levels of both empowerment dimensions, suggesting a potential link between maternal empowerment and improved child nutritional status. In contrast, northern regions of the country displayed the most concerning nutritional indicators in 2011. However, all northern regions demonstrated improvement by 2016. Notably, Tigray achieved a substantial reduction in stunting, decreasing from 51.7% to 39.7%, representing one of the most significant regional gains during the period.

The Somali region presented a particularly complex pattern. Despite consistently ranking among the lowest in women's capability scores, it maintained relatively low stunting prevalence, which declined from 32.8% to 27.2% between survey waves. At the same time, Somali experienced substantial improvement in women's decision‐making authority, which rose from 0.47 to 0.72. This divergence highlights the potential for specific dimensions of empowerment to influence child nutritional outcomes even in contexts of limited overall capability.

To further validate and quantify the regional patterns observed in the descriptive analysis, we conducted statistical subgroup analysis examining the strength of empowerment‐stunting associations within each region. This analysis revealed substantial heterogeneity in the associations between women's empowerment indices and child stunting across Ethiopia's 11 regions, providing statistical rigor to the descriptive regional variations previously identified. Statistically significant negative associations between the capability index and stunting were observed in four regions: Addis Ababa (log odds = –0.36, 95% CI: –0.53 to –0.20, *p* < 0.001), Somali (–0.25, 95% CI: –0.41 to –0.09, *p* < 0.01), Southern Nations, Nationalities, and Peoples' Region (SNNPR) (–0.23, 95% CI: –0.34 to –0.11, *p* < 0.001), and Gambela (–0.18, 95% CI: –0.31 to –0.05, *p* < 0.01). The strongest protective association was found in Addis Ababa, where a one‐unit increase in the capability index was associated with approximately 30% lower odds of stunting. In the remaining seven regions, associations between the capability index and stunting were not statistically significant, with effect sizes ranging from near null in Tigray (–0.04, 95% CI: –0.15 to 0.07) to moderately protective in Afar (–0.19, 95% CI: –0.40 to 0.01). Benishangul‐Gumuz was the only region to exhibit a positive, though nonsignificant, coefficient (0.018, 95% CI: –0.13 to 0.17).

In contrast, the decision‐making index demonstrated less consistent patterns across regions. Only Afar showed a statistically significant negative association between autonomy and stunting (–0.15, 95% CI: –0.24 to –0.06, *p* < 0.01), indicating that greater decision‐making power was associated with lower odds of stunting in this region. No other region showed statistically significant results, and the direction of effects varied widely. For example, the coefficient for Harari was positive (0.16, 95% CI: –0.003 to 0.33), whereas Addis Ababa showed a negative, though nonsignificant, association (–0.20, 95% CI: –0.47 to 0.07). Detailed estimates, including log odds coefficients, standard errors, confidence intervals, p‐values, and significance levels, are presented in Table 4.

**Table 4:** Logistic regression results by region, showing associations between women's empowerment indices and child stunting.

Region	Capability index					Decision making index				
	Coef.	SE	95% CI		*p*‐value	Coef.	SE	95% CI		*p*‐value
Amhara	−0.11	0.065	−0.24	0.02	0.089	−0.03	0.055	−0.14	0.08	0.58
Addis Ababa	−0.36***	0.084	−0.53	−0.20	< 0.001	−0.20	0.14	−0.47	0.07	0.15
Harari	−0.12	0.077	−0.27	0.03	0.12	0.16	0.084	−0.003	0.33	0.054
Somali	−0.25**	0.081	−0.41	−0.09	0.002	0.06	0.042	−0.02	0.14	0.14
Benishangul Gumuz	0.018	0.077	−0.13	0.17	0.82	−0.05	0.051	−0.15	0.05	0.33
Oromia	−0.10	0.058	−0.22	0.01	0.08	0.05	0.042	−0.03	0.14	0.21
SNNPR	−0.23***	0.059	−0.34	−0.11	< 0.001	0.02	0.041	−0.06	0.10	0.65
Tigray	−0.04	0.058	−0.15	0.07	0.49	0.04	0.057	−0.08	0.15	0.52
Afar	−0.19	0.11	−0.40	0.01	0.07	−0.15**	0.046	−0.24	−0.06	0.001
Gambela	−0.18**	0.065	−0.31	−0.05	0.005	0.02	0.057	−0.09	0.13	0.72
Dire Dawa	−0.17	0.088	−0.34	0.00	0.056	−0.05	0.080	−0.20	0.11	0.56

*Note:* Coefficients represent log‐odds estimates from logistic regression models predicting child stunting (1 = stunted, 0 = not stunted). Models adjust for survey wave, mother's age category, child's age category, birth order category, and standardized household wealth index. Significance levels:

* *p* < 0.05

** *p* < 0.01

*** *p* < 0.001.

These findings suggest that the capability dimension of women's empowerment may serve as a more consistent and robust predictor of child stunting across regional contexts in Ethiopia compared to decision‐making autonomy. This pattern highlights the potential importance of structural factors linked to capability, such as women's access to education, health services, and economic resources, in shaping child nutritional outcomes, rather than interpersonal or household‐level autonomy alone.

## 4 Discussion

Our analysis of the associations between two dimensions of women's empowerment (capabilities and decision‐making authority) and child stunting using the 2011 and 2016 EDHS data reveals four key findings. First, women's capabilities (education, literacy, media access) showed consistent negative associations with child stunting, while decision‐making authority showed no significant association. The differential effects between capability and decision‐making indices suggest that these empowerment dimensions operate through distinct pathways in influencing child nutrition. Second, substantial regional heterogeneity exists in both empowerment dimensions and their associations with stunting. Third, the interaction between capabilities and decision‐making was statistically significant, though the interaction model showed only marginal improvement in fit over the main effects specification. Fourth, temporal analysis revealed that while overall stunting decreased between waves, the strength of the capability‐stunting association remained consistent over time.

These findings both align with and contrast with previous research on women's empowerment and child nutrition. The negative association between capabilities and stunting is consistent with Shroff et al.'s (2009) findings about maternal autonomy being inversely associated with stunting in India. However, the finding that decision‐making authority showed no significant relationship with stunting contrasts with Yaya et al. (2020), who found significant associations between all three empowerment measures and child nutrition across 30 sub‐Saharan African countries. Similarly, the results partially contradict Mekonnen et al. (2021), who identified decision‐making as significantly associated with reduced stunting in Ethiopia using only 2016 EDHS data and the survey‐based women's emPowERment (SWPER) index approach with three domains (attitude to violence, social independence, and decision‐making).

The contrasting findings may reflect methodological differences: Mekonnen et al. (2021) used a single cross‐sectional wave and standardized SWPER methodology, while our study employed two survey waves with custom capability‐decision indices validated through factor analysis. Additionally, Wassie et al. (2024) studied only rural northwest Ethiopia with 582 mother‐child pairs and five empowerment dimensions (household decision‐making, education, cash earnings, house/land ownership, community group membership), finding significant associations for overall empowerment but mixed results for individual decision‐making components. The substantial regional heterogeneity documented here strongly validates Richardson. (2018) emphasis on context‐specific measurement approaches while extending Wassie et al.'s (2024) work on empowerment and stunting by demonstrating how these relationships vary across Ethiopia's diverse regions.

Stunting prevalence decreased from 42.3% to 36.4% nationally between 2011 and 2016, demonstrating Ethiopia's progress amid sustained economic growth averaging 9.1% annually (World Bank 2020). However, the persistence of high stunting rates (36.4%) despite economic advancement suggests complex barriers to translating macroeconomic gains into equitable nutritional improvements.

To better understand these patterns, we first examined the underlying structure of women's empowerment dimensions. The factor analysis presented in Appendix Table A3 empirically validates the conceptual distinction between capabilities (educational attainment, literacy status, media exposure) and decision‐making authority (healthcare decisions, household purchases, mobility choices) as separate empowerment dimensions. Between 2011 and 2016, divergent trends emerged in empowerment dimensions: decision‐making authority increased substantially (from 0.62 0.70 to 0.73 0.78) while capability scores remained relatively stable (from 0.17 to 0.16) (see Apendix Table A12). This pattern is particularly striking given that 71% of Ethiopian women report participation in household decisions, yet only 20% have completed primary education (CSA & ICF, 2016). This differs from findings in Bangladesh, where Holland and Rammohan (2019) documented concurrent improvements in both dimensions.

The notable decline in media access scores (from 0.30 to 0.17) may reflect complex changes in Ethiopia's information landscape during this period. While political factors, including the 2009 antiterrorism proclamation and increased media restrictions, potentially contributed to reduced traditional media access (Freedom House 2016; Human Rights Watch 2015), technological shifts may have also played a critical role. During 2011–2016, Ethiopia experienced rapid expansion of mobile phone infrastructure, with multiple sources indicating significant growth in mobile subscriptions (World Bank 2024). This shift toward mobile technology potentially altered information consumption patterns as social media platforms became increasingly important for information dissemination, particularly during political unrest (Workneh 2021). However, the DHS media exposure questions focus exclusively on newspapers, radio, and television, potentially missing this transition to digital platforms accessed via mobile devices. This measurement limitation could explain the paradox of declining traditional media access scores amid expanding digital connectivity.

The consistent protective associations of capabilities across both survey‐weighted (Appendix Tables A9 and A10) and hierarchical models (Table 2 and Appendix Table A11) suggest that fundamental skills—such as education, literacy, and information access—play critical roles in optimal feeding practices and healthcare decisions. Conversely, the null association with decision‐making authority indicates that formal household power without corresponding capabilities may be insufficient for improving child nutrition outcomes. The temporal stability of these relationships (Appendix Table A12) suggests that capability‐nutrition pathways remained robust despite social and economic changes during this period. The statistical associations between capabilities and child stunting were consistent across model specifications. In both survey‐weighted and hierarchical models, the capability index showed a significant negative association with stunting (*β* = −0.141, *p* < 0.001 in hierarchical model), while the decision‐making index showed no significant association. The capability‐wave interaction term in our interaction model (Appendix Table A10) was not statistically significant (*β* = −0.094, *p* = 0.082), indicating that the association between capability scores and stunting remained stable between 2011 and 2016. However, the significant capability‐decision interaction (Appendix Table A11) (Table 2) reveals the capability‐decision interaction (Appendix Table A11) was significant (*β* = 0.055 0.050, *p* = 0.007 0.011), indicating that the protective association of capabilities diminishes as decision‐making authority increases, suggesting these empowerment dimensions may operate as substitutes rather than complements. The unexpected positive direction of the decision‐making coefficient in some models could potentially reflect complex household dynamics where formal authority without corresponding capabilities creates tensions or might suggest reverse causality where women in households with malnourished children gain more decision‐making responsibilities as a coping mechanism.

Several plausible mechanisms may also be associated with the observed relationship between higher capabilities and lower stunting rates. Women with higher education and literacy may have better access to nutritional information, improved health‐seeking behaviors, and enhanced ability to implement optimal feeding practices, consistent with findings by Cunningham et al. (2019) in Nepal. In contrast, formal decision‐making authority without corresponding knowledge may be insufficient to show associations with improved child nutrition outcomes.

The models revealed important patterns among control variables. Child age showed the expected epidemiological pattern, with increasing stunting risk across age groups from 0 to 24 months, consistent with established evidence (Karlsson et al. 2023). The observed negative coefficients indicate lower stunting prevalence in younger children, reflecting the typical developmental pattern where stunting accumulates with age. Household wealth demonstrated a nonlinear relationship with stunting, with significant negative associations only in the richer and richest quintiles.

The regional analysis reveals substantial variations in empowerment measures and their associations with child stunting. The Somali region presents a particularly intriguing case: despite maintaining low capability scores, it experienced a substantial stunting reduction (32.8%–27.2%), which occurred alongside significant gains in decision‐making authority (0.47– 0.72). These patterns may be associated with the implementation of nutrition interventions through the Productive Safety Net Programme (PSNP) and ONE WASH National Programme, humanitarian assistance following the 2011 Horn of Africa drought crisis, and potentially distinctive nutritional pathways associated with pastoralist lifestyles. In contrast, Addis Ababa shows the lowest stunting rates alongside the highest capability scores, patterns that may be associated with better infrastructure, higher female literacy, and different gender norms. Despite improvements in measured empowerment dimensions, Amhara region's persistently high stunting rates suggest that additional factors such as agricultural practices, dietary diversity, and feeding practices may play important roles in nutritional outcomes. These regional variations align with Richardson. (2018) emphasis on enabling environments but contrast with findings of more uniform empowerment effects documented by Nikiema and Kponou (2024).

The contradictory trends between mean HAZ and stunting prevalence observed in Gambela, Harari, and Dire Dawa may reflect distributional changes around the stunting threshold (−2 SD). As demonstrated by Leroy and Frongillo (2019), when intervention efforts prioritize children near the cutoff point, they can shift the distribution without necessarily changing overall means. This threshold effect underscores the importance of examining both continuous HAZ measures and binary stunting classifications when evaluating nutrition‐related outcomes.

This study has several methodological and measurement limitations that constrain the interpretation of its findings. Most notably, its cross‐sectional design and observational data preclude any causal inference. While national sample sizes were substantial, regional subsamples varied considerably, limiting statistical power to detect smaller associations in certain areas.

The empowerment indices used face challenges in constructing validity. The capability index, for example, assigns total weight to education 50% and literacy 30% based on factor loadings, potentially reflecting educational attainment more than broader capabilities. These indicators (education, literacy) are also typically measured with greater precision than self‐reported decision‐making variables, which are vulnerable to social desirability bias. Women may overreport decision‐making involvement to align with perceived interviewer expectations, possibly weakening observed associations.

Media exposure measures overlook digital media usage, which may result in an underestimation of modern information access. Although both empowerment dimensions exhibit strong internal consistency, about one‐third of the variance in empowerment remains unexplained by the measured indicators, underscoring the complexity of quantifying empowerment through standardized surveys. Additionally, certain regions, particularly pastoral areas, exhibited significantly higher anthropometric data missingness (Afar: 23.1%; Somali: 20.9%), potentially introducing bias in regional comparisons.

## 5 Conclusion and Future Research Directions

These findings have important implications for nutrition and gender programming in Ethiopia and similar contexts. The consistent association between capability measures and lower stunting rates suggests that investments in women's education, literacy, and information access may be associated with better child nutrition outcomes, while interventions focused solely on enhancing women's household authority without addressing capability deficits may show limited associations with outcomes. These findings align with Hoddinott et al.'s (2013) demonstration that nutrition interventions yield benefit‐cost ratios ranging from 3.6 to 48, potentially making capability‐focused women's empowerment programs among the most cost‐effective development investments available. The substantial regional heterogeneity underscores the need for regionally tailored approaches that consider unique sociocultural contexts.

The results directly inform Ethiopia's current policy landscape, including the Seqota Declaration (FDRE Ministry of Health 2015) and National Nutrition Program II (Federal Democratic Republic of Ethiopia 2016), which aims to end child undernutrition by 2030. Following a successful Innovation Phase in 40 woredas (2016–2020), the government launched the Expansion Phase in 240 woredas (2021–2025). As of January 2024, coverage expanded to 700 woredas within this phase, requiring significant investment to achieve its goals (FDRE Ministry of Health 2024). The initiative will continue with the National Scale‐up Phase (2026–2030), when interventions will be implemented across the entire country. Our findings suggest this ongoing scale‐up should prioritize capability‐building interventions over decision‐making focused programs through: (1) integrating adult literacy training with nutrition counseling through health extension workers, (2) allocating additional resources to pastoral regions (Afar, Somali) where capability scores remain below 0.10, and (3) modifying monitoring frameworks to track education and information access alongside traditional decision‐making indicators.

With 148.1 million stunted children globally (UNICEF, W., WB. 2023), these findings may inform progress toward multiple Sustainable Development Goals, particularly SDG 2 (Zero Hunger) and SDG 5 (Gender Equality). The observed capability‐nutrition associations directly relate target 2.2—ending all forms of malnutrition, including stunting by 2025, while the empowerment findings may inform targets 5.5 (women's full participation in decision‐making) and 5.a (women's equal rights to economic resources) (United Nations 2015). The regional variations identified are consistent with Sachs et al. (2022) evidence on subnational implementation strategies, while persistent capability gaps in regions like Afar and Somali highlight challenges in meeting SDG 4.5 on eliminating educational disparities.

Our findings validate the multidimensional nature of women's empowerment building on Alkire et al. (2015) measurement framework. This study extends Sen. (1999) and Kabeer. (1999) theoretical frameworks by providing empirical evidence of how different dimensions of empowerment are associated with child nutrition outcomes in Ethiopia. Our analysis of regional variations advances methodological approaches to studying women's empowerment in diverse sociocultural settings. The findings have practical implications for nutrition programming, where interventions often prioritize household decision‐making authority without sufficient attention to building foundational capabilities (Jones et al. 2020).

Future research should prioritize panel data approaches that follow the same households over time to better understand potential causal pathways linking empowerment dimensions to child nutrition. Mixed methods approaches could also clarify the mechanisms underlying these relationships across Ethiopia's diverse contexts.

### 5.1 Recommendations

Based on our findings, Ethiopian nutrition programming must prioritize women's capability development (specifically functional literacy, education completion, and information access) through: (1) integrating functional adult literacy with nutrition counseling; (2) regional tailoring, particularly for areas like Somali and Afar with low capabilities; (3) multi‐sectoral programming linking nutrition interventions with capability enhancement; and (4) disaggregated monitoring frameworks to track empowerment dimensions. Policymakers must allocate resources toward multi‐sectoral programming linking education and nutrition services rather than standalone empowerment interventions, with intensive capability building in pastoral regions where scores remain below 0.10. International organizations can support Ethiopia's 2030 nutrition targets by financing capability‐focused interventions in high‐burden regions using evidence‐based targeting strategies, building fundamental capabilities alongside decision‐making interventions with attention to local contexts and constraints.

## Author Contributions

Seyoum Teffera conceptualized the research, performed statistical data analysis, and drafted the manuscript. Eva Berde contributed to research design, manuscript writing and revision. Zerihun Yohannes provided critical insights into conceptualization of the research, data interpretation and contributed to manuscript editing. Sandor Remsei assisted with the literature review and data interpretation. All authors read and approved of the final manuscript.

## Conflicts of Interest

The authors declare no conflicts of interest related to this study.

## Data Availability

The data that support the findings of this study are available on request from the corresponding author. The data are not publicly available due to privacy or ethical restrictions.

## Endnotes

1 Initially, we intended to include a broader data set to strengthen our analysis. However, after careful evaluation, we excluded certain waves due to significant limitations. The 2005 EDHS had severe data quality issues, with 54.8% missing anthropometric data compared to 10%–15% in later waves. Additionally, it used the older NCHS/WHO growth reference from 1977 (Hamill et al. 1979) rather than the 2006 WHO Child Growth Standards (WHO 2006), and only 45.2% of surveyed women had children under 5 years. The 2019 Mini‐DHS was excluded since it completely lacked women's decision‐making variables despite having better anthropometric data quality. As shown in Appendix Table 1, the proportion of valid anthropometric data increased substantially from 39.3% in 2005%–82.5% in 2011% and 83.2% in 2016Concurrently, the proportion of flagged cases declined from 13.1% to 8.29% and 1.36%, respectively, thereby validating our methodological choices.

## 1

***Appendix Models***

**Model A1: Survey‐weighted main effects model**:

$$\mathrm{logit}(P(\mathrm{stunting}))=\alpha +\beta_{1}\mathrm{Capability}+\beta_{2}\mathrm{Decision}+\beta_{3}\mathrm{Wave}+\gamma \;\mathrm{Controls}$$

**Model A2: Survey‐weighted wave interaction model**:

$$\mathrm{logit}(P(\mathrm{stunting}))=\alpha +\beta_{1}\mathrm{Capability}+\beta_{2}\mathrm{Decision}+\beta_{3}\mathrm{Wave}+\beta_{4}(\mathrm{Capability}\times \mathrm{Wave})+\beta_{5}(\mathrm{Decision}\times \mathrm{Wave})+\mathrm{\gamma Controls}$$

**Model A3: Hierarchical main effects model**:

$$\mathrm{logit}(P({\mathrm{stunting}}_{\mathrm{ir}}))=\alpha +\beta_{1}\mathrm{Capability}+\beta_{2}\mathrm{Decision}+\beta_{3}\mathrm{Wave}+\gamma \;\mathrm{Controls}+u_{r}$$

where $\beta_{4}$ and $\beta_{5}$ represent the interaction effects between capability/decision‐making and wave.

Table A1

**Table A1:** Data quality and survey characteristics across EDHS waves.

Characteristic	2005	2011	2016	2019
Sample characteristics				
Initial sample size	9861	11,654	10,641	5753
Women with under‐5 children (%)	45.2	89.9	91.1	—
Children with anthropometric data (*n*)	4455	10,480	8977	5126
Children with valid anthropometric data (*n*)	3873	9611	8855	5045
Data quality				
Valid anthropometric data (%)	39.3	82.5	83.2	88.2
Flagged cases (%)	13.1	7.5	1.1	0.98
Missing data (%)	54.8	10.1	15.6	10.9
Survey design				
Number of strata	22	23	25	21
Number of primary sampling units (PSUs)	540	596	643	305
Growth standards	NCHS/WHO 1977	WHO (2006)	WHO (2006)	WHO (2006)
Decision‐making variables (n)	9220	10,779	10,008	0

**Table A2:** Missing data analysis by survey wave.

Characteristic	Valid anthropometric cases	Cases without valid measurements
2011 Wave		
Sample size (*n*)	9,611	2,043
Capability score (mean)	0.162	0.161
Decision score (mean)	0.677	0.649
Urban residence (%)	16.1	21.6
2016 Wave		
Sample size (*n*)	8,855	1,786
Capability score (mean)	0.177	0.164
Decision score (mean)	0.779	0.778
Urban residence (%)	18.4	19.5

**Appendix** Tables A3, A4, A5 **: Validation of Empowerment Measures**

The indices were validated using factor analysis and reliability tests in three carefully executed stages. First, we conducted exploratory factor analysis using a polychoric correlation matrix to account for the ordinal nature of several indicators (Holgado‐Tello et al. 2010). This analysis employed promax rotation with two specified factors, based on our theoretical framework. We assessed model fit comprehensively using chi‐square tests, factor loadings, and explained variance (Fabrigar et al. 1999). The factor analysis provided strong support for a two‐dimensional structure of women's empowerment, with capability and decision‐making emerging as distinct constructs.

The analysis demonstrated improved explanatory power, with total variance increasing from 54.4% in 2011%–62.6% in 2016 (Factor 1: 32.3%, Factor 2: 30.3%). Factor loadings showed clear discrimination between dimensions. Capability components loaded strongly on Factor 1, with education (0.865), literacy (0.900), and media access (0.615) showing distinct patterns. Decision‐making items loaded clearly on Factor 2, with healthcare (0.834), purchases (0.751), and mobility (0.747) decisions forming a coherent dimension. Model fit improved from 2011 ($\chi^{2}$ = 2.94, df = 4, *p* = 0.568) to 2016 $\chi^{2}$ = 6.93, df = 4, *p* = 0.140), suggesting increasing structural clarity in empowerment measurement.

**Table A3:** Factor analysis results by wave.

Variable	2011 Factor 1	2016 Factor 1	2011 Factor 2	2016 Factor 2
Education	0.857	0.865	—	—
Literacy	0.911	0.900	—	—
Media access	0.545	0.615	—	—
Healthcare decisions	—	—	0.775	0.834
Purchase decisions	—	—	0.686	0.751
Mobility decisions	—	—	0.571	0.747
Variance explained	31.0%	32.3%	23.4%	30.3%
Total variance	54.4%	62.6%	—	—
Model fit (*χ*²)	2.94 (*p* = 0.568)	6.93 (*p* = 0.140)	—	—

In the second stage, we evaluated measurement reliability through multiple approaches. Internal consistency was assessed via Cronbach's alpha, using the standard threshold of 0.7 for acceptable reliability (DeVellis & Thorpe, 2021). We conducted detailed examination of inter‐item correlations within each dimension to verify cohesive measurement, while also analyzing between‐dimension correlations to confirm discriminant validity. Response consistency was carefully evaluated through test‐retest comparisons using a 10% random subsample.

Both indices demonstrated robust internal consistency. The capability index showed high reliability with Cronbach's α values of 0.783 in 2011 and 0.820 in 2016, accompanied by strong inter‐item correlations. Within the capability dimension, education and literacy scores showed the strongest association (*r* = 0.766 in 2011; *r* = 0.779 in 2016), while education‐media (*r* = 0.478 in 2011; *r* = 0.533 in 2016) and literacy‐media correlations (*r* = 0.512 in 2011; *r* = 0.556 in 2016) demonstrated moderate relationships.

The decision‐making index displayed notable improvement in reliability metrics over time. Cronbach's α strengthened substantially from 0.710 in 2011–0.819 in 2016. Inter‐item correlations showed consistent improvement across waves, particularly for health‐purchase decisions (*r* = 0.521–0.629) and health‐mobility decisions (*r* = 0.440–0.621). The relationship between purchase and mobility decisions demonstrated the most substantial strengthening (*r* = 0.386–0.561), suggesting increasing coherence in decision‐making patterns over time.

**Table A4:** Psychometric properties, 2011–2016.

Property	2011	2016
Internal consistency		
Capability index (α)	0.783	0.820
Decision index (α)	0.710	0.819
Inter‐domain correlations		
Within capability	0.41–0.76	0.43–0.76
Within decision	0.38–0.49	0.47–0.56
Between indices	0.15–0.20	0.14–0.19
Missing data (%)		
Education	0.0	0.0
Literacy	3.3	1.8
Media exposure	0.0	0.0
Healthcare decisions	7.6	6.0
Purchase decisions	7.6	6.0
Mobility decisions	7.6	6.0
Response consistency	0.87	0.89

To comprehensively assess robustness, we conducted detailed sensitivity analyses using alternative weighting schemes for combining the capability and decision‐making indices. These analyses tested six distinct weighting combinations, ranging from heavily favoring capabilities (70% capability, 30% decision‐making) to heavily favoring decision‐making (20% capability, 80% decision‐making).

The sensitivity analyses demonstrated robust findings across different weighting specifications. The baseline weighting scheme (40:60 capability:decision ratio) produced balanced distributions with moderate negative skewness that increased from −0.379 in 2011 to −0.782 in 2016. More extreme weightings yielded predictable distributional shifts: capability‐dominant weights (70:30) produced pronounced positive skewness (0.903 in 2011, 0.825 in 2016), while decision‐dominant weights (20:80) resulted in strong negative skewness (−0.659 in 2011, −1.232 in 2016). Importantly, the temporal trends in empowerment remained consistent across all weighting schemes, supporting the validity of our main findings.

**Table A5:** Sensitivity analysis of alternative weighting schemes for combined empowerment index, 2011–2016.

Weighting scheme	2011 (Capability:Decision)			2016 (Capability:Decision)		
	Mean	SD	Skewness	Mean	SD	Skewness
Base (40:60)	0.470	0.262	−0.379	0.536	0.253	−0.782
Equal (50:50)	0.418	0.240	−0.072	0.475	0.237	−0.335
Capability‐heavy (60:40)	0.366	0.225	0.371	0.414	0.227	0.238
Decision‐heavy (30:70)	0.521	0.287	−0.562	0.596	0.274	−1.073
Capability‐dominant (70:30)	0.314	0.215	0.903	0.353	0.225	0.825
Decision‐dominant (20:80)	0.573	0.315	−0.659	0.657	0.299	−1.232

To assess regional statistical power, we conducted a MDE analysis. Urban regions with smaller cluster sizes demonstrated greater statistical sensitivity. Notably, Addis Ababa showed the lowest MDEs across both waves (capability: 0.980–1.13; decision‐making: 0.693–0.742; HAZ: 0.695–0.703), reflecting the efficiency gains from its compact sampling design. In contrast, regions with larger cluster sizes like Somali required larger effects for detection (capability: 1.70–1.75; decision‐making: 0.850‐–1.08; HAZ: 0.855–0.965), likely due to greater within‐cluster correlation.

The MDE patterns also showed temporal changes between waves. The 2016 survey generally achieved improved sensitivity for decision‐making and anthropometric measures compared to 2011, though capability MDEs remained similar. This suggests the 2016 survey design may have better accounted for clustering effects in these domains.

**Table A6:** Minimum detectable effects by region and survey wave.

	2011 MDEs			2016 MDEs		
Region	Capability	Decision	HAZ	Capability	Decision	HAZ
Tigray	1.47	0.965	0.875	1.52	0.787	0.791
Afar	1.58	1.02	0.917	1.66	0.825	0.830
Amhara	1.40	0.933	0.849	1.40	0.758	0.761
Oromia	1.57	1.02	0.916	1.71	0.839	0.844
Somali	1.70	1.08	0.965	1.75	0.850	0.855
Benishangul‐Gumuz	1.51	0.984	0.889	1.57	0.799	0.804
SNNPR	1.54	1.00	0.903	1.58	0.804	0.808
Gambela	1.42	0.941	0.856	1.43	0.763	0.767
Harari	1.32	0.895	0.820	1.40	0.757	0.761
Addis Ababa	0.980	0.742	0.703	1.13	0.693	0.695
Dire Dawa	1.35	0.910	0.831	1.34	0.742	0.746

**Table A7:** Missing data patterns by domain and survey wave.

Domain/variable	2011 *n* (%)	2016 *n* (%)
**Anthropometric**		
Total missing	2,043 (17.5)	1,786 (16.8)
– Flagged values	869 (7.5)	122 (1.1)
– True missing	1,174 (10.1)	1,664 (15.6)
Capability		
All indicators	0 (0.0)	0 (0.0)
Decision‐making		
All indicators	875 (7.5)	633 (5.9)
Control variables		
Child age	1174 (10.1)	945 (8.9)
All others	0 (0.0)	0 (0.0)

**Table A8:** Regional sample distribution and anthropometric data availability, Ethiopia 2011–2016.

	2011		2016		Missingness	
Region	*N*	%	*n*	%	*n*	%
Tigray	1202	10.3	1033	9.7	8.2	8.5
Afar	1130	9.7	1062	10.0	13.7	23.1
Amhara	1294	11.1	977	9.2	9.1	12.4
Oromia	1761	15.1	1581	14.9	10.8	18.3
Somali	1027	8.8	1505	14.1	12.5	20.9
Benishangul‐Gumuz	1020	8.7	879	8.3	10.2	15.8
SNNPR	1614	13.8	1277	12.0	8.0	11.6
Gambela	851	7.3	714	6.7	19.3	22.8
Harari	659	5.7	605	5.7	17.9	24.5
Addis Ababa	400	3.4	461	4.3	8.0	14.1
Dire Dawa	696	6.0	547	5.1	8.3	13.6

**Table A9:** Association between women's empowerment dimensions and child stunting in Ethiopia (2011–2016).

Variable	Coefficient (SE)	aOR	95% CI	*p*‐value
Intercept	0.052 (0.245)	1.05	(0.65, 1.71)	0.832
Main predictors				
Capability index	−0.154 (0.034)	0.86	(0.80, 0.92)	< 0.001***
Decision index	0.018 (0.026)	1.02	(0.97, 1.07)	0.483
Wave (2016 vs. 2011)	−0.204 (0.058)	0.82	(0.73, 0.92)	< 0.001***
Maternal age (ref: 15–19)				
20–24	−0.307 (0.146)	0.74	(0.55, 0.98)	0.035*
25–29	−0.350 (0.159)	0.70	(0.51, 0.96)	0.028*
30–34	−0.338 (0.163)	0.71	(0.52, 0.98)	0.039*
35–39	−0.353 (0.172)	0.70	(0.50, 0.98)	0.040*
40–44	−0.488 (0.187)	0.61	(0.42, 0.89)	0.009**
45–49	−0.522 (0.222)	0.59	(0.38, 0.92)	0.019*
Child age (ref: 24–59 m)				
0–5 m	−1.616 (0.093)	0.20	(0.17, 0.24)	< 0.001***
6–11 m	−1.391 (0.093)	0.25	(0.21, 0.30)	< 0.001***
12–23 m	−0.292 (0.055)	0.75	(0.67, 0.83)	< 0.001***
Birth order (ref: Fifth + )				
First	0.163 (0.109)	1.18	(0.95, 1.46)	0.133
Second	−0.001 (0.094)	1.00	(0.83, 1.20)	0.995
Third	−0.012 (0.084)	0.99	(0.84, 1.16)	0.882
Fourth	−0.015 (0.076)	0.99	(0.85, 1.15)	0.848
Wealth (ref: Poorest)				
Poorer	0.060 (0.083)	1.06	(0.90, 1.25)	0.469
Middle	−0.083 (0.076)	0.92	(0.79, 1.07)	0.272
Richer	−0.198 (0.088)	0.82	(0.69, 0.98)	0.024*
Richest	−0.455 (0.109)	0.63	(0.51, 0.78)	< 0.001***
Residence (ref: Urban)				
Rural	0.083 (0.132)	1.09	(0.84, 1.41)	0.527
Region (ref: Addis Ababa)				
Tigray	0.605 (0.148)	1.83	(1.37, 2.45)	< 0.001***
Afar	0.462 (0.157)	1.59	(1.17, 2.16)	0.003**
Amhara	0.743 (0.150)	2.10	(1.57, 2.82)	< 0.001***
Oromia	0.326 (0.153)	1.39	(1.03, 1.87)	0.033*
Somali	−0.095 (0.153)	0.91	(0.67, 1.23)	0.533
Benishangul‐Gumuz	0.538 (0.154)	1.71	(1.27, 2.32)	< 0.001***
SNNPR	0.421 (0.151)	1.52	(1.13, 2.05)	0.006**
Gambela	−0.236 (0.154)	0.79	(0.58, 1.07)	0.126
Harari	0.105 (0.150)	1.11	(0.83, 1.49)	0.484
Dire Dawa	0.381 (0.145)	1.46	(1.10, 1.95)	0.009**

*Note:* Dependent variable is stunting status (1 if stunted, 0 otherwise). Survey weights applied. Quasibinomial logistic regression accounts for complex survey design. Significance levels:

Abbreviations: aOR = adjusted odds ratio, CI = confidence interval.

* *p* < 0.05

** *p* < 0.01

*** *p* < 0.001.

**Table A10:** Survey‐weighted interaction model of child stunting.

Variable	Coefficient (SE)	aOR	95% CI	*p*‐value
Intercept	0.071 (0.244)	1.07	(0.67, 1.72)	0.771
Capability Index	−0.108 (0.041)	0.90	(0.83, 0.97)	0.008**
Decision Index	0.022 (0.036)	1.02	(0.95, 1.10)	0.545
Wave (2016)	−0.210 (0.058)	0.81	(0.72, 0.91)	< 0.001***
Maternal age (ref: 15–19)				
20–24	−0.306 (0.146)	0.74	(0.55, 0.98)	0.037*
25–29	−0.346 (0.160)	0.71	(0.52, 0.97)	0.031*
30–34	−0.336 (0.164)	0.71	(0.52, 0.98)	0.041*
35–39	−0.353 (0.172)	0.70	(0.50, 0.98)	0.040*
40–44	−0.485 (0.187)	0.62	(0.43, 0.89)	0.010**
45–49	−0.519 (0.222)	0.59	(0.38, 0.92)	0.020*
Child age (ref: 24–59 m)				
0–5 m	−1.615 (0.093)	0.20	(0.17, 0.24)	< 0.001***
6–11 m	−1.389 (0.093)	0.25	(0.21, 0.30)	< 0.001***
12–23 m	−0.291 (0.055)	0.75	(0.67, 0.83)	< 0.001***
Birth Order (ref: Fifth + )				
First	0.169 (0.109)	1.18	(0.96, 1.46)	0.120
Second	0.004 (0.094)	1.00	(0.83, 1.21)	0.969
Third	−0.012 (0.084)	0.99	(0.84, 1.16)	0.888
Fourth	−0.013 (0.076)	0.99	(0.85, 1.15)	0.860
Wealth (ref: Poorest)				
Poorer	0.045 (0.084)	1.05	(0.89, 1.23)	0.588
Middle	−0.093 (0.076)	0.91	(0.78, 1.06)	0.220
Richer	−0.211 (0.087)	0.81	(0.68, 0.96)	0.016*
Richest	−0.468 (0.110)	0.63	(0.51, 0.78)	< 0.001***
Residence (ref: Urban)				
Rural	0.080 (0.132)	1.08	(0.84, 1.40)	0.543
Region (ref: Addis Ababa)				
Tigray	0.600 (0.148)	1.82	(1.36, 2.44)	< 0.001***
Afar	0.450 (0.156)	1.57	(1.16, 2.13)	0.004**
Amhara	0.738 (0.149)	2.09	(1.56, 2.80)	< 0.001***
Oromia	0.316 (0.152)	1.37	(1.02, 1.85)	0.038*
Somali	−0.111 (0.154)	0.90	(0.66, 1.21)	0.471
Benishangul‐Gumuz	0.532 (0.153)	1.70	(1.26, 2.30)	< 0.001***
SNNPR	0.414 (0.151)	1.51	(1.12, 2.04)	0.006**
Gambela	−0.247 (0.155)	0.78	(0.58, 1.06)	0.110
Harari	0.098 (0.149)	1.10	(0.82, 1.48)	0.512
Dire Dawa	0.376 (0.144)	1.46	(1.10, 1.93)	0.009**
Interaction terms				
Capability × Wave	−0.094 (0.054)	0.91	(0.82, 1.01)	0.082
Decision × Wave	−0.006 (0.052)	0.99	(0.90, 1.10)	0.901

*Note:* Dependent variable is stunting status. Survey weights applied. Significance levels:

* *p* < 0.05

** *p* < 0.01

*** *p* < 0.001.

**Table A11:** Hierarchical model of child stunting in Ethiopia (2011–2016).

Variable	Coefficient (SE)	aOR	95% CI	*p*‐value
Intercept	0.163 (0.154)	1.18	(0.87, 1.60)	0.289
Main predictors				
Capability index	−0.153 (0.021)	0.86	(0.83, 0.90)	< 0.001***
Decision index	0.002 (0.016)	1.00	(0.97, 1.03)	0.921
Wave (2016 vs. 2011)	−0.165 (0.033)	0.85	(0.80, 0.91)	< 0.001***
Maternal age (ref: 15‐19)				
20–24	−0.083 (0.090)	0.92	(0.77, 1.10)	0.359
25–29	−0.094 (0.094)	0.91	(0.76, 1.09)	0.314
30–34	−0.148 (0.100)	0.86	(0.71, 1.05)	0.139
35–39	−0.126 (0.104)	0.88	(0.72, 1.08)	0.226
40–44	−0.181 (0.115)	0.83	(0.67, 1.04)	0.115
45–49	−0.386 (0.144)	0.68	(0.51, 0.90)	0.007**
Child age (ref: 24–59 m)				
0–5 m	−1.638 (0.061)	0.19	(0.17, 0.22)	< 0.001***
6–11 m	−1.410 (0.061)	0.24	(0.22, 0.27)	< 0.001***
12–23 m	−0.246 (0.039)	0.78	(0.72, 0.84)	< 0.001***
Birth order (ref: Fifth + )				
First	0.093 (0.069)	1.10	(0.96, 1.25)	0.180
Second	−0.119 (0.058)	0.89	(0.79, 0.99)	0.039*
Third	−0.059 (0.053)	0.94	(0.85, 1.05)	0.267
Fourth	−0.014 (0.051)	0.99	(0.89, 1.09)	0.782
Wealth (ref: Poorest)				
Poorer	−0.055 (0.050)	0.95	(0.86, 1.05)	0.274
Middle	−0.096 (0.050)	0.91	(0.82, 1.01)	0.056
Richer	−0.256 (0.052)	0.77	(0.70, 0.86)	< 0.001***
Richest	−0.486 (0.073)	0.61	(0.53, 0.71)	< 0.001***
Residence (ref: Urban)				
Rural	0.106 (0.067)	1.11	(0.98, 1.27)	0.111
Random effects				
Region variance	0.084	—	—	—

*Note:* aOR = adjusted Odds Ratio; CI = Confidence Interval. Dependent variable is stunting status. Hierarchical model with random intercepts for regions. Significance levels:

* *p* < 0.05

** *p* < 0.01

*** *p* < 0.001.

**Table A12:** Survey‐weighted empowerment dimensions in Ethiopia, 2011–2016.

	2011		2016		
Dimension	Mean	SE	Mean	SE	Change
Capability index	0.17	0.007	0.16	0.007	−0.01
Education	0.12	0.010	0.14	0.012	0.02
Literacy	0.17	0.017	0.18	0.018	0.01
Media access	0.30	0.016	0.17	0.015	−0.13
Decision‐making index	0.70	0.009	0.78	0.009	0.08
Healthcare decisions	0.72	0.010	0.79	0.009	0.07
Large purchases	0.64	0.010	0.76	0.009	0.12
Mobility	0.76	0.009	0.82	0.009	0.06
